# A Dual Polybasic Motif Determines Phosphoinositide Binding and Regulation in the P2X Channel Family

**DOI:** 10.1371/journal.pone.0040595

**Published:** 2012-07-11

**Authors:** Louis-Philippe Bernier, Dominique Blais, Éric Boué-Grabot, Philippe Séguéla

**Affiliations:** 1 Department of Neurology and Neurosurgery, Montreal Neurological Institute, Alan Edwards Centre for Research on Pain, McGill University, Montréal, Québec, Canada; 2 Institut des Maladies Neurodégénératives, Université Bordeaux Segalen, CNRS UMR 5293, Bordeaux, France; University of Tokyo, Japan

## Abstract

Phosphoinositides modulate the function of several ion channels, including most ATP-gated P2X receptor channels in neurons and glia, but little is known about the underlying molecular mechanism. We identified a phosphoinositide-binding motif formed of two clusters of positively charged amino acids located on the P2X cytosolic C-terminal domain, proximal to the second transmembrane domain. For all known P2X subtypes, the specific arrangement of basic residues in these semi-conserved clusters determines their sensitivity to membrane phospholipids. Neutralization of these positive charges disrupts the functional properties of the prototypical phosphoinositide-binding P2X4 subtype, mimicking wortmannin-induced phosphoinositide depletion, whereas adding basic residues at homologous positions to the natively insensitive P2X5 subtype establishes *de nov*o phosphoinositide-mediated regulation. Moreover, biochemical evidence of in vitro P2X subunit-phospholipid interaction and functional intracellular phosphoinositide-binding assays demonstrate that the dual polybasic cluster is necessary and sufficient for regulation of P2X signaling by phospholipids.

## Introduction

ATP-gated P2X receptor channels play significant roles in pain transduction, neuro-immune interactions and inflammatory response therefore understanding their regulation mechanisms is critical. Plasma membrane phosphoinositides (PIP_n_) are anionic phospholipids that act as functional regulators of many types of ion channels. They are necessary cofactors for activation or desensitization of various channels, including transient receptor potential (TRP) channels [1], [2], inward rectifier K^+^ (Kir) channels [3], [4] and voltage-gated KCNQ channels [5]. Most ATP-gated P2X receptor subtypes are potentiated by intracellular PIP_n_. P2X1, P2X2, P2X3, P2X4 and P2X7 are functionally sensitive to PIP_n_ [6], [7], [8], [9], however, P2X5 was found to be PIP_n_-insensitive [10].

In P2X subunits, the few residues shown to be implicated in PIP_n_-mediated regulation are located in the C-terminal domain, which is also involved in subunit trafficking, phosphorylation, heteromerization and multi-receptor crosstalks [11], [12], [13], [14].

Although no consensus PIP_n_ binding site exists among membrane proteins, analysis of the PIP_n_-binding region of PH domain-containing proteins points to the necessary presence of basic amino acids interacting with the anionic headgroup of PIP_n_ [15]. The identity of PIP_n_-binding domains in ion channels in particular has been even more elusive, with only a few putative residues identified as involved in the interaction, all on the cytoplasmic side of the membrane [for review [16]]. Although direct protein-phospholipid binding was demonstrated for several families of PIP_n_-sensitive channels, no common motif can predict an effective interaction.

Here, we demonstrate that P2X channel subunits bind to PIP_n_ via two clusters of positively charged residues located in the proximal C-terminal domain. The specific arrangement of basic and acidic amino acids found in these semi-conserved clusters predicts the PIP_n_-sensitivity of all known P2X subtypes. By mutating the prototypical PIP_n_-sensitive P2X4 and PIP_n_-insensitive P2X5 subtypes, we provide functional and biochemical evidence that a dual cluster motif in the proximal C-terminal domain is necessary and sufficient for the regulation of P2X receptor channels by PIP_n_.

## Results

### A PIP_n_-binding Site in P2X C-terminal Domains

**Figure 1:**
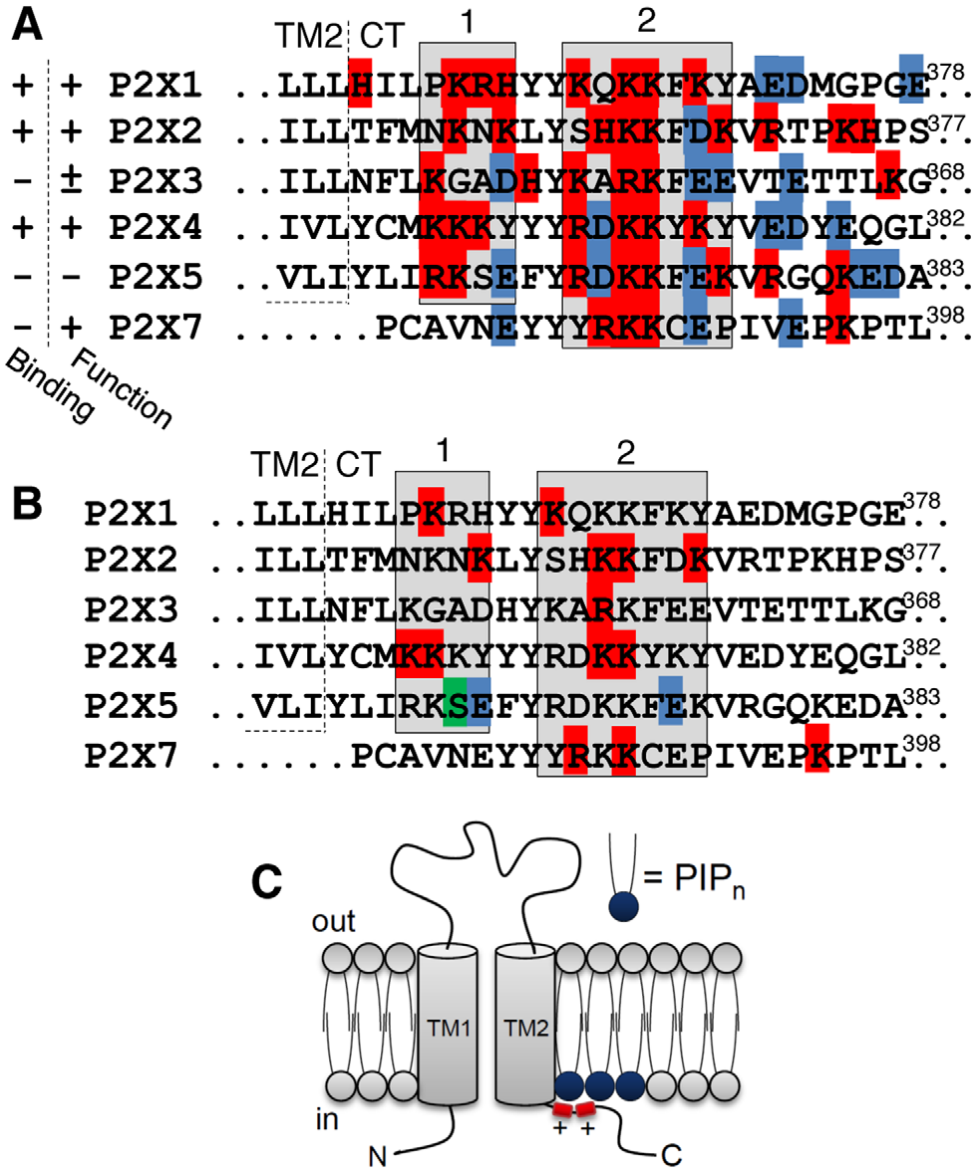
The proximal C-terminal domain of P2X subunits contains a semi-conserved PIP_n_-binding motif. A) Sequence alignment of rat P2X C-termini proximal to the TM2 domain showing the two polybasic clusters (shaded area, 1 and 2). The left column summarizes, for each subunit, the presence (+) or absence (−) of binding of the GST-fusion C-terminal domain to PIP_n_ in PIP strip assays. The second column shows the presence (+) or absence (−) of modulation by PIP_n_ in functional assays. Basic residues are shown in red and acidic residues in blue. B) Sequences showing residues that were reported (here or previously) to be involved in PIP_n_ regulation. Basic residues in red, acidic residues in blue and an uncharged serine in green. C) Schematic representation of the topology of a P2X subunit showing binding of two positively charged amino acid clusters to membrane-bound PIP_n_.

Several studies have demonstrated functional modulation of P2X receptors by PIP_n_ as well as direct PIP_n_ binding to the C-terminal domain of various P2X subunits (Figure 1A, left columns) [6], [7], [8], [9], [10], [17]. P2X1, P2X2 and P2X4 subunits directly bind PIP_n_, whereas P2X3 and P2X5 do not. Hence, we analyzed their respective C-terminal sequences and found that PIP_n_ binding correlates with the net positive charge of two polybasic amino acid clusters (Figure 1, shaded areas 1 and 2). P2X1/2/4 contain 6–7 basic residues (lysine, arginine or histidine) in these two clusters and a maximum of one negatively-charged residue (aspartic or glutamic acid). On the other hand, the non PIP_n_-binding P2X3 and P2X5 subunits contain 5 and 6 basic residues, respectively, but also 3 acidic residues disrupting the global positive charge of the clusters. We therefore hypothesized that the dual cluster’s charge is responsible for the affinity of the P2X C-terminus to PIP_n_. Interestingly, a conserved hydrophobic tyrosine residue is located between the two clusters, and could also be involved in the interaction. This cytosolic region located 3 residues from the second transmembrane domain (TM2) region lies in close proximity to the plasma membrane where electrostatic interactions with the negative phosphate head groups of the membrane-anchored PIP_n_ can take place.

A closer look at the single residues that have, in this report or previously, been demonstrated to be involved in PIP_n_-mediated regulation shows they are all (except one in P2X7) located within the two polybasic clusters (Fig. 1B). No evidence of binding has been found on the N-terminal domain of any P2X subunit. The P2X6 subtype was excluded from our study since it does not form functional homomeric receptors [18].

### A Dual Polybasic Cluster Motif is Necessary for PIP_n_-binding and P2X4 Channel Regulation

**Figure 2:**
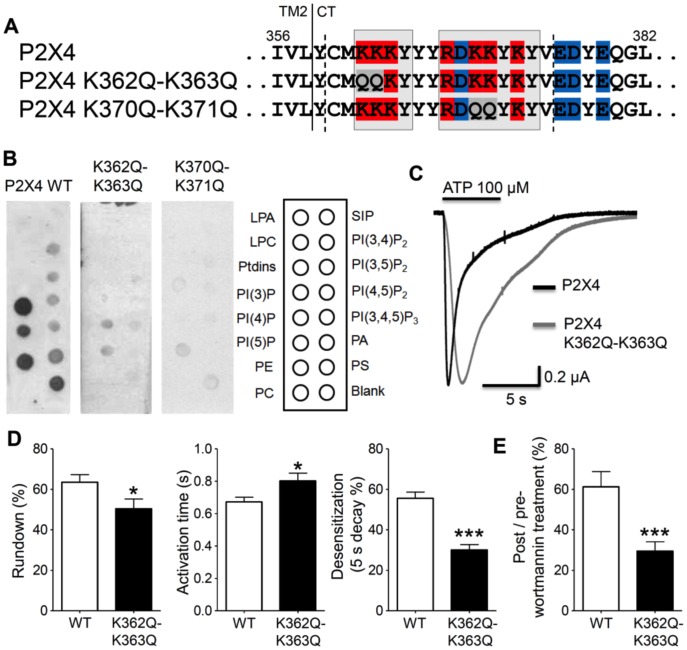
Requirement of two polybasic clusters in the PIP_n_-regulated P2X4 subtype. A) Sequence of the P2X4 C-terminus showing lysine to glutamine mutations disrupting the positive charge of the first or second cluster (basic residues in red, acidic in blue, neutral mutations in grey). B) The GST construct containing the P2X4 C-terminal domain C360-V375 binds to several PIP_n_ including PIP_2_ and PIP_3_. Mutating the basic lysine residues K362 and K363, or K370 and K371 into neutral glutamine leads to a loss of binding to PIP_n_ (n = 3–6). C) Representative ATP-activated P2X4 current traces obtained on P2X4-expressing *Xenopus* oocytes showing the slower activation and desensitization rates induced by the K362Q-K363Q mutation decreasing PIP_n_-binding affinity. D) Quantitative analysis of the functional changes induced by the K362Q-K363Q mutation on P2X4 current rundown (left), activation (middle) and desensitization (right). A larger rundown between agonist applications is observed with the mutant than with the WT (2^nd^/1^st^ application: WT: 63.5±3.8%, mutant: 50.4±4.9%, n = 10–11). The mutant P2X4 channel shows a slower activation rate (10–90% rise time: WT: 0.67±0.03 s, mutant: 0.80±0.05 s, n = 70–80) and a slower desensitization rate (5-second decay %: WT: 55.6±3.1%, mutant: 30.1±2.6%, n = 55–61). E) Wortmannin-induced PIP_n_ depletion leads to a stronger inhibition of P2X4 current amplitude in the K362Q-K363Q mutant than in WT (post/pre-treatment: WT: 61.3±7.5%, mutant: 29.5±4.6%, n = 30–50). *: p<0.05; ***: p<0.001.

We reported that P2X4 is a prototypical PIP_n_-dependent P2X subtype, being tightly regulated via direct binding to PIP_2_ and PIP_3_ [8]. We therefore aimed to neutralize the PIP_n_-binding site by mutating key lysine residues. We found that neutralizing the charge of either of the two clusters, by mutating lysines 362 and 363 or lysines 370 and 371 into neutral glutamines, leads to a loss of PIP_n_ binding in an *in vitro* binding assay where a GST-fusion protein coding for a 16-amino acid sequence (Figure 2A,B) is applied to various PIP_n_. The lysine-to-glutamine mutations performed on residues 362 and 363 also induced significant changes in the P2X4 channel activity. Expressed in the *Xenopus* oocyte expression system, the P2X4 mutant with lower PIP_n_-binding affinity displayed a stronger current rundown upon repeated ATP applications as well as slower activation and desensitization current phases (Figure 2C,D), all these effects mimicking those brought by pharmacological PIP_n_ depletion [8]. The K362Q-K363Q mutant receptor was more strongly inhibited by wortmannin-induced PIP_n_ depletion than the wild-type (WT) receptor (Figure 2E), due to its lower affinity to PIP_n_. The mutation targetting the second basic cluster (K370Q-K371Q) could not be tested functionally as P2X4 channels with mutations on residue 371 are non-functional due to the role of conserved lysine 371 in receptor trafficking [11].

### P2X1 and P2X7 Binding to PIP_n_ is Consistent with the Dual Polybasic Cluster Model

**Figure 3:**
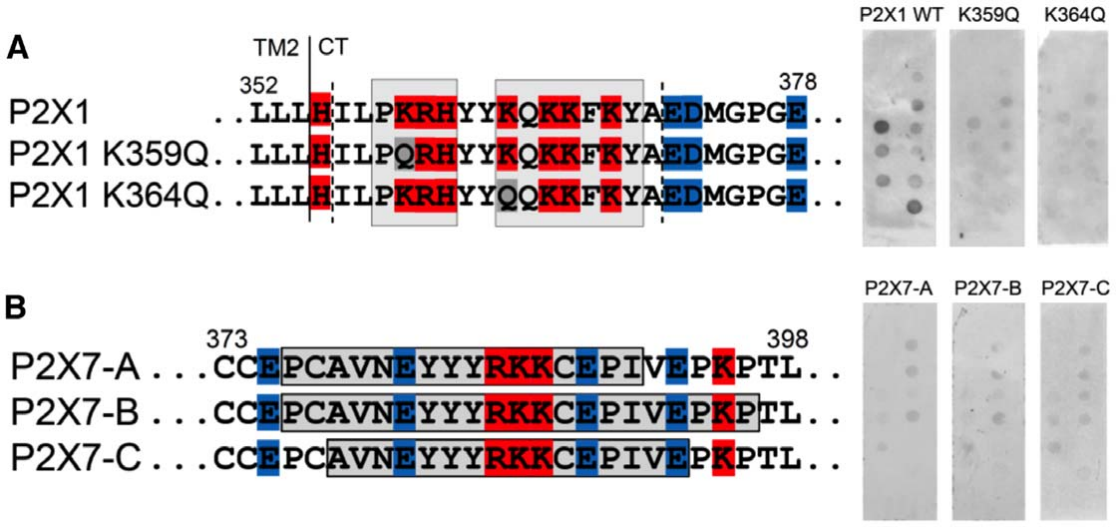
Requirement of two polybasic clusters for PIP_n_-binding in P2X1 and P2X7. A) The GST construct containing the WT P2X1 C-terminal domain (L352-E378) (basic residues in red, acidic in blue, neutral mutations in grey) binds various PIP_n_ on a phospholipid strip assay, whereas disrupting the positive charge of the first or second polybasic cluster with K359Q and K364Q mutations suppresses binding (n = 3). B) The absence of two polybasic clusters in the C-terminus of P2X7 prevents its binding to PIP_n_ on a phospholipid strip assay (n = 3). Shown in grey boxes are various GST-fusion peptides generated.

For the P2X1 subtype, the results that we have previously reported are consistent with our model, in that mutating the K359 residue in the first cluster suppressed in vitro binding to PIP_n_, and induced a PIP_n_-depleted like current phenotype [7]. To confirm that both clusters are involved in the interaction with PIP_n_, we neutralized the charge in the second cluster via a lysine-to-glutamine mutation on residue 364. This also induced a loss of binding in a phospholipid strip assay (Figure 3A), confirming that both clusters are necessary for the P2X1 C-terminus to bind PIP_n_.

The P2X7 subtype was also analyzed, but no direct binding was found in our biochemical binding assay using C-terminal peptides of various length (Figure 3B). The absence of binding is likely due to the presence of only one polybasic cluster in the P2X7 C-terminus. Nevertheless, a previous report demonstrates through a mutational study that specific amino acids are involved in PIP_n_ modulation of P2X7 [17], suggesting a more complex binding mechanism, likely due to the presence of an additional 18-residue long sequence between the cluster and TM2.

### Generation of De Novo PIP_n_-regulation in the P2X5 Subtype

**Figure 4:**
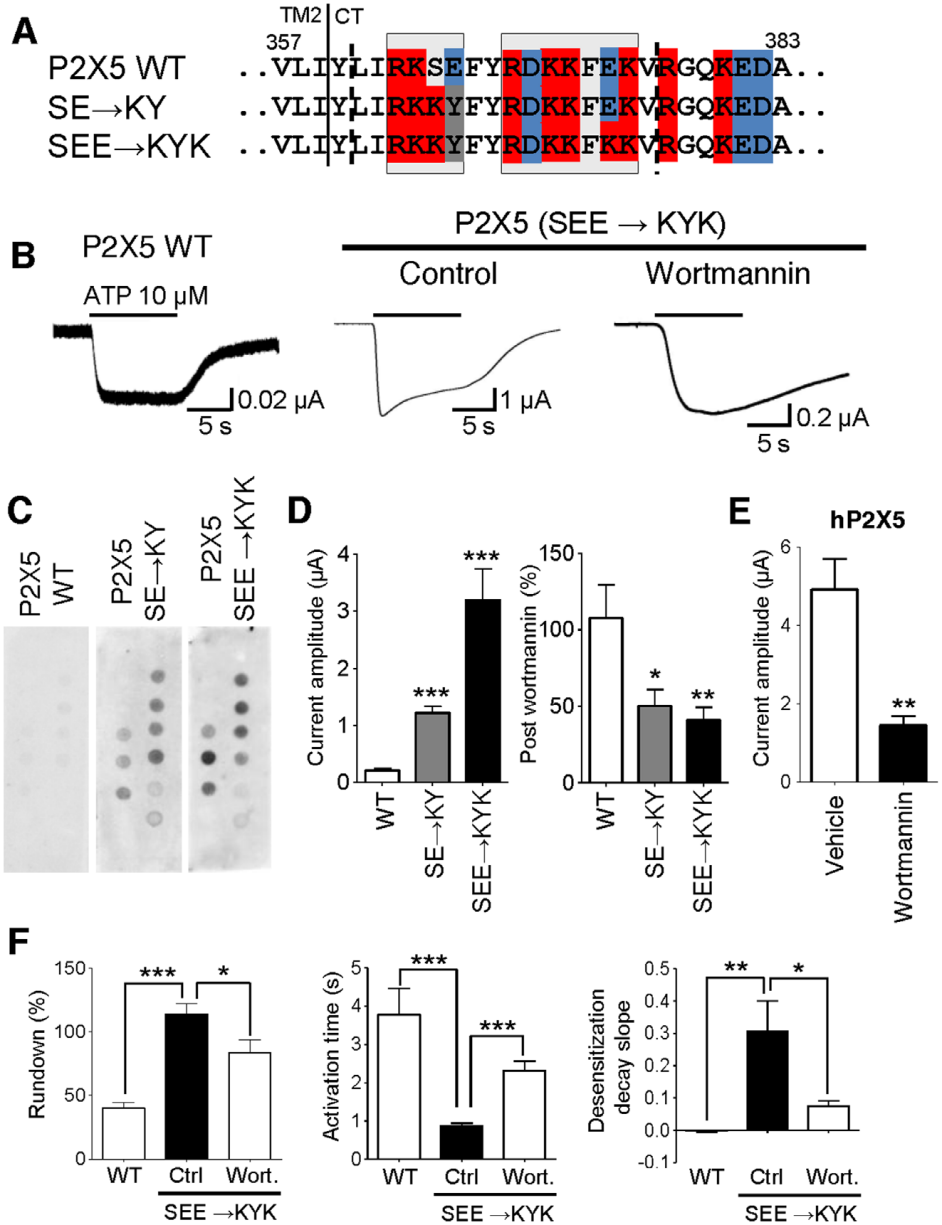
Mutations creating two polybasic clusters on the P2X5 C-terminus lead to PIP_n_ binding and a PIP_n_-regulated current phenotype. A) Sequence of the P2X5 C-terminus showing mutations adding positive charges to one (S365K-E366Y, SE→KY) or two clusters (S365K-E366Y-E374K, SEE→KYK) (basic residues in red, acidic in blue, neutral mutations in grey). B) Representative ATP-activated current traces recorded in *Xenopus* oocytes expressing P2X5 WT or SEE→KYK mutant in control and wortmannin conditions. C) The GST construct containing the WT P2X5 proximal C-terminal domain L361-V376 does not bind to PIP_n_. Adding positive residues to one amino acid cluster with the SE→KY mutation induces binding to several PIP_n_. Creating a second positive cluster with the SEE→KYK mutation increases PIP_n_ binding (n = 3–6). D) Quantitative data showing the SE→KY and SEE→KYK gain-of-binding mutations lead to increased current amplitude in rP2X5-expressing *Xenopus* oocytes (left graph; rP2X5 WT: 0.21±0.04 µA, SE→KY: 1.22±0.11 µA, SEE→KYY: 3.19±0.55 µA, n = 13–25). The gain-of-binding mutants are sensitive to intracellular PIP_n_ levels as wortmannin-induced PIP_n_ depletion leads to a decrease in current amplitude (right graph, post/pre-treatment amplitude; WT: 107.5±21.8%, SE→KY: 50.3±10.7%, SEE→KYY: 40.9±8.4%, n = 4–17). E) Human P2X5 channel currents are significantly inhibited by PIP_n_ depletion (vehicle = 4.90±0.79 µA; post-wortmannin = 1.44±0.24 µA, n = 7–10). F) Differences in current rundown, activation rate and desensitization rate between WT P2X5 and SEE→KYK mutant under control and PIP_n_-depletion conditions. The current rundown between successive applications measured with the WT P2X5 is prevented by gain-of-binding mutations, and is partially restored after a wortmannin treatment of the mutant (2^nd^/1^st^ application: WT: 40.0±4.4%, mutant control: 113.9±8.0%, mutant wortmannin: 83.7±9.8%, n = 5–10). The mutant P2X5 channel shows a faster current activation compared to WT, and it is slowed by PIP_n_ depletion (10–90% rise time: WT: 3.77±0.69 s, mutant control: 0.86±0.08 s, mutant wortmannin: 2.31±0.25 s, n = 7–8). The gain-of-binding P2X5 mutant current desensitizes faster than WT, and its desensitization rate is slowed by PIP_n_ depletion (decay slope: WT: −0.001±0.006, mutant control: 0.31±0.09, mutant wortmannin: 0.07±0.02, n = 7–8). *: p<0.05; **: p<0.01; ***: p<0.001.

To verify if the presence of C-terminal polybasic clusters is sufficient for PIP_n_ regulation of P2X receptor channels, we chose the natively PIP_n_-insensitive P2X5 subunit and examined the effect of adding basic residues to its C-terminus (Figure 4A,B). In the phospholipid binding assay, adding positive charges to the cluster proximal to TM2 by mutating residues 365 and 366 induced binding to several PIP_n_. Also, mutating a negatively-charged glutamic acid into a lysine in the second cluster enhanced binding, thereby showing that PIP_n_ binding can be obtained via negative-to-positive mutations in the twin clusters (Figure 4C). We then analyzed the functional effect of that mutation by recording from the S365K-E366Y-E374K mutant in the *Xenopus* oocyte expression system: the mutant P2X5 receptor generated currents ∼15 times larger than the WT upon 10 µM ATP activation. Adding basic residues to the first cluster only (S365K-E366Y) also led to significantly larger currents than the WT. Whereas WT P2X5 is unaffected by wortmannin treatment, both PIP_n_-binding mutants were strongly inhibited by wortmannin-induced PIP_n_ depletion, suggesting that PIP_n_ binding is responsible for the current amplitude increase induced by the C-terminal mutations (Figure 4B,D). WT P2X5 channels display a marked current rundown upon repeated activation; such a feature was absent in the triple mutant, but could be restored after pharmacological PIP_n_ depletion (Figure 4F). Also, the activation rate of the P2X5 current was faster in the PIP_n_-binding mutant than in the WT, as was the desensitization rate. Both properties were restored towards WT levels after wortmannin treatment, confirming the PIP_n_-sensitivity of the mutant receptor channel.

The human P2X5 ortholog has an arginine residue on position 365 and has been shown to evoke currents of much larger amplitude than its rat homolog [19], we therefore verified if PIP_n_ play a role in this difference. Wortmannin-induced PIP_n_ depletion significantly reduced the hP2X5 current amplitude (Figure 4E), indicating that interspecies differences exist in terms of functional PIP_n_ regulation of P2X5 channels and confirming the importance of the positive charges found in the proximal polybasic cluster.

### P2X4 C-terminal Peptides Compete for Intracellular PIP_n_ and Induce a PIP_n_-depletion Current Phenotype

**Figure 5:**
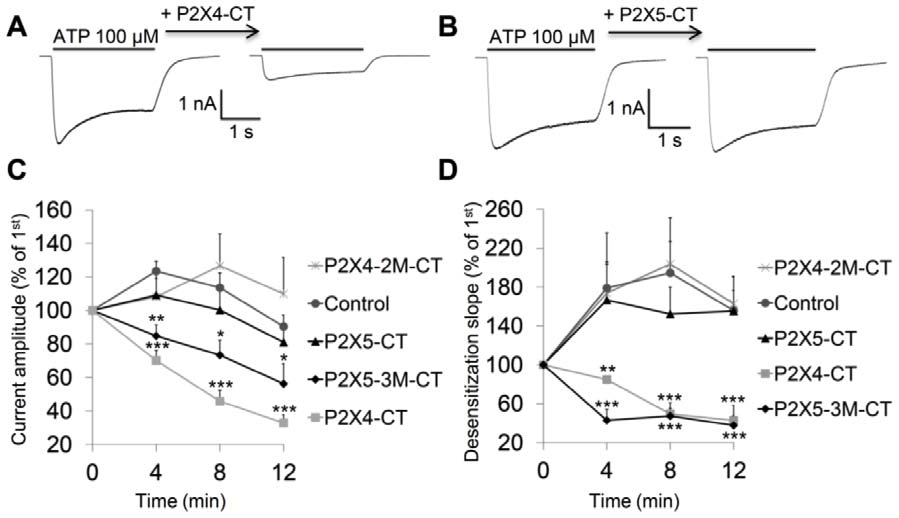
P2X C-terminal peptides compete with P2X channels for binding to intracellular PIP_n_. A) Representative traces of patch-clamp recordings showing that intracellular injection of the P2X4 C-terminal (-CT) peptide leads to a rundown of the P2X4 current in HEK293 cells by competing for intracellular PIP_n_. B) Intracellular injection of the P2X5-CT peptide does not affect the P2X4 current phenotype. C) Effect on P2X4 current amplitude of injection of peptides from the P2X4 WT, P2X4 K362Q-K363Q (2M), P2X5 WT or P2X5 S365K-E366Y-E374K (3M) C-terminus. D) Competition for PIP_n_ binding from P2X4-CT or P2X5-3M-CT peptide injection leads to a slower desensitization of the P2X4 current. Values were normalized to the initial recording value obtained immediately after whole-cell configuration was obtained (n = 4–5; *, p<0.05; **, p<0.01; ***, p<0.001, each group compared to control).

To confirm that P2X C-terminal polybasic clusters bind to PIP_n_ in a cytoplasmic environment, we performed an intracellular PIP_n_-binding competition assay. HEK293 cells transiently expressing P2X4 were recorded in whole-cell patch-clamp configuration, and various GST fusion proteins containing the P2X C-termini peptides (16 amino acid-long, P2X4: C360-V375, P2X5: L361-V376) were added to the intracellular milieu. When PIP_n_-binding P2X4 C-terminus peptides were introduced through the patch pipette, a strong rundown of the ATP-mediated P2X4 current as well as a strong decrease in desensitization rate were observed, suggesting that the P2X4 C-terminal peptide competes for intracellular PIP_n_ binding, inducing a PIP_n_-depletion current phenotype (Figure 5A,C,D). Peptides coding for the P2X4 K362Q-K363Q mutant C-terminal domain did not induce any change in the current phenotype as compared to a control GST peptide injection, indicating an inability to bind intracellular PIP_n_. Reciprocally, the WT P2X5 C-terminal peptide had no effect on both functional parameters measured (Figure 5B,C,D) due to its low PIP_n_-binding affinity. Strong interactions between the P2X5 S365K-E366Y-E374K mutant peptides and PIP_n_ led to rundown and slower desensitization of the P2X4 currents.

## Discussion

Membrane PIP_n_ regulate the activity of a wide variety of ion channels, and the mechanism of interaction between these important membrane proteins and the anionic phospholipids draws lots of attention. We show here that the modulation of P2X channel function by PIP_n_ is predicted by the subunit’s ability to bind to the negative inositol triphosphate head group of the lipid via two adjacent clusters of basic amino acids located on the C-terminal domain. Binding of PIP_n_ to the clusters present in P2X1, P2X2 and P2X4 likely leads to a conformational change in the C-terminus, a domain highly involved in functional regulation of the channel.

The P2X3, P2X5 and P2X7 subunits lack this microdomain and therefore do not directly interact with PIP_n_. While the absence of this microdomain renders P2X5 channel activity insensitive to PIP_n_, P2X3 and P2X7 are functionally modulated by PIP_n_ [6], [17], strongly indicating an indirect regulation. A mechanism in which a PIP_n_-binding partner protein acts as a regulatory subunit has been proposed for TRPV1, where *phosphoinositide interacting regulator of TRP* (Pirt) is necessary for PIP_n_-mediated enhancement of the channel activity [20]. Pirt, a membrane protein which binds PIP_2_ via a cluster of basic residues on its C-terminus, also complexes with TRPV1 to link both molecules. A similar interaction was observed in the case of NMDA receptors, where the cytosolic tails of the NR1 or NR2B subunits bind α-actinin, an actin-crosslinking protein. α-actinin also binds membrane PIP_2_ and modifies the NMDA receptor’s intracellular tail conformation to promote channel opening [21]. A similar mechanism could underlie the indirect PIP_n_-dependent regulation of P2X3 and P2X7, but the nature of the partner involved remains to be eludicated. Interestingly, P2X7 forms a signalling complex with various proteins that includes α-actinin [22], which could link the P2X7 C-terminal tail to PIP_n_.

Proteins bind PIP_n_ via multiple contacts that require the contribution of multiple amino acids [16], hence we analyzed the general charge of the P2X C-terminal domain, and found that the 13-amino acid sequence containing both clusters has a predicted isoelectric point of 10.4 to 10.8 for P2X1/2/4/5, but that P2X3 and P2X7 have lower predicted values of 9.2 and 8.5, respectively. This could explain the lack of direct PIP_n_ binding in these two subunits. However, PIP_n_-sensitive P2X1/2/4 and PIP_n_-insensitive P2X5 subunits have similar isoelectric points, suggesting that PIP_n_ interaction is not only determined by the global charge of the structure, but depends on the specific spatial arrangement of charged amino acids. Although the C-terminal domains of P2X4 and P2X5 contain a similar number of basic residues, the presence of 3 acidic residues distributed within the two clusters of P2X5 disrupts the electrostatic pattern required for effective PIP_n_ affinity.

While single cationic residues involved in subunit-PIP_n_ interaction have been identified for most PIP_n_-dependent channels, clusters of basic residues were found in the TRP box of TRPM8 channels as well as in the carboxy-terminal tail of Kv7 channels [23], [24]. It was also reported that a conserved sequence in the juxtamembrane C-terminus of Kir 1.1 and Kir 2.1 takes part in the protein-phospholipid interaction [25]. X-ray crystal structure modelling confirmed that the equivalent structure in Kir6.2 is part of the PIP_2_-binding pocket that includes 3 other basic residues [26]. Furthermore, the crystallization of Kir 2.2 and of GIRK2 in the presence of short-chain PIP_2_ directly shows that PIP_2_-binding can induce significant conformational changes that modulate the channel function [27], [28]. The recently published crystal structure of a truncated zebrafish P2X4.1 receptor [29] unfortunately does not contain the N- and C-terminal tails, therefore additional work will be needed to obtain structural information on this intracellular regulatory motif and on the exact conformational effect of PIP_n_-binding.

Another property common to the primary sequence of most known PIP_n_-binding pockets is the presence of at least one aromatic residue [15]. Interestingly, a fully conserved tyrosine residue lies between the two clusters forming the proposed P2X PIP_n_-binding motif. Mutating this residue on the P2X1 (Y363Q) and P2X4 (Y367Q) C-terminal sequence did not affect the binding to PIP_n_ in *in vitro* overlay assays (data not shown). However, it may play a structural role in the secondary or tertiary conformation of the dual cluster domain, a possibility that could not be tested functionally as mutating this conserved residue induces a loss of function [11].

The gating mechanism underlying ion conduction in P2X channels has been extensively studied in recent years. It is believed that the gate is located between residues 340 to 347 (nomenclature for zP2X4.1) in the TM2 domain, and that opening of the channel triggers rearrangement of the TM2 helices that reveals access to even deeper parts of the pore a few residues away from the cytoplasmic tail [29], [30], [31]. The PIP_n_-binding region therefore lies in close proximity to the gating machinery of P2X receptor channels, likely impacting on the open/closed transition through conformational changes.

Results obtained on the P2X4 and P2X5 subunits not only enhance our understanding of the PIP_n_-binding site of P2X receptors, but also demonstrate the importance of PIP_n_ in the functional regulation of the channels. Disrupting the PIP_n_ affinity of P2X4 led to major changes in the current phenotype, similar to what was seen in other PIP_n_-binding P2X subtypes [7], [9]. Moreover, we were able for the first time to induce a PIP_n_-binding phenotype through single mutations in the otherwise PIP_n_-insensitive P2X5 subtype, demonstrating that the polybasic clusters motif is sufficient for PIP_n_ binding and functional regulation. The high amplitude currents obtained with the gain-of-binding mutant suggest that the small size of currents mediated by the WT rat P2X5 in several expression systems [10], [32] is due to its low PIP_n_ affinity. Altogether, our results indicate that membrane PIP_n_ contribute to the full expression of P2X receptor channel function.

Interestingly, it was shown that the human and chicken P2X5 receptors give rise to currents that are significantly larger, and desensitize faster than their rat counterpart [19], [33]. Analysis of their C-terminal sequence shows that human P2X5 has a basic arginine residue on position 365, instead of a neutral serine found in the rat sequence, and that chicken P2X5 has a neutral asparagine on position 366, instead of a negatively-charged glutamic acid in the rat sequence. Our results demonstrate that a neutral-to-basic mutation on residue 365 and an acidic-to-neutral mutation on residue 366 induces a PIP_n_-binding phenotype in the rat P2X5. We also show that the human P2X5 channel is regulated by PIP_n_ as pharmacological depletion of intracellular PIP_n_ induced dramatic changes to its current phenotype. It has to be noted that on position 375, at the extremity of the second polybasic cluster, a lysine is found in the rat sequence instead of a glutamic acid in the human and chicken orthologs. This suggests that the first cluster plays a preponderent role in PIP_n_ binding, in agreement with the gain-of-binding S365K-E366Y mutation performed on rat P2X5. Since mutations increasing the PIP_n_-binding affinity of P2X5 have a drastic effect on its ion channel function, it is likely that these differences in C-terminal sequence account for the high degree of variability of P2X5 phenotypes observed among vertebrate species.

Identification of the molecular determinants of PIP_n_-protein interactions in the P2X family confirmed the intrinsic and essential nature of PIP_n_ regulation of P2X channel activity. Knowing that intracellular PIP_n_ levels are controlled by a wide array of ubiquitous pathways such as G_q_-coupled receptor-induced phospholipase C hydrolysis of PIP_2_ or receptor tyrosine kinase activation of PI3K, the P2X-PIP_n_ regulatory mechanism is likely involved in multi-receptor crosstalks. Our predictive model unifies various data obtained on PIP_n_-regulation of P2X receptors in physiological and pathological contexts and also provides useful insights on PIP_n_-regulation mechanisms of other ion channels.

## Materials and Methods

### Two-electrode Voltage-clamp Recordings in Xenopus Oocytes

Oocytes were removed from *Xenopus laevis* frogs as described [8] before intranuclear microinjection of 1 ng plasmid DNA coding for rat P2X4 (WT or K362Q-K363Q), rat P2X5 (WT, S365K-E366Y or S365K-E366Y-E374K) or human P2X5 (kind gift from Dr Alan North). Oocytes were then incubated in Barth’s solution containing 1.8 mM CaCl_2_ at 19°C for 24 to 72 h before electrophysiological recordings. Two-electrode voltage-clamp recordings (Vhold = −60 mV for P2X4, −120 mV for P2X5) were performed using glass pipettes (1–3 MΩ) filled with 3 M KCl solution. The external Ringer’s solution, pH 7.4, contained (in mM): 115 NaCl, 5 NaOH, 2.5 KCl, 1.8 CaCl_2_, and 10 HEPES. Membrane currents were recorded using a Warner OC-725B amplifier (Warner Instruments) and digitized at 1 kHz. For PIP_n_-depletion experiments, oocytes were incubated in 10 µM wortmannin for 1–2 hours prior to recording. Each series of recordings consisted of three successive applications of ATP (10 or 100 µM), with a 4-minute wash in Ringer’s solution between each application.

### Whole-cell Patch-clamp Recordings on HEK293 Cells

HEK293 cells (ATCC) were cultured in Dulbecco’s modified Eagle’s medium and 10% heat-inactivated fetal bovine serum (Invitrogen) containing penicillin and streptomycin supplemented with G-418 (250 µg/ml). The cells were transiently co-transfected with pEGFP and pCDNA3-rP2X4 using Polyfect (Qiagen) according to the manufacturer’s instructions. Transfected cells were used for electrophysiological recordings 48 h after transfection. Whole-cell recordings (*V*_hold_ = −60 mV) were performed using pipettes filled with internal solution, pH 7.2, containing (in mM): 120 K-gluconate, 1 MgCl_2_, 5 EGTA and 10 HEPES. The recording solution, pH 7.4, comprised (in mM):140 NaCl, 5 KCl, 2 CaCl_2_, 2 MgCl_2_, 10 HEPES and 10 glucose. GST-fusion peptides (GST control, P2X4-CT, P2X4 K362Q-K363Q-CT, P2X5-CT or P2X5 S365K-E366Y-E374K-CT) were dissolved in the internal pipette solution to a 1 µM concentration. Membrane currents were recorded using an Axopatch 200B amplifier and digitized at 500 Hz with a Digidata 1330 interface (Axon Instruments). Agonists were dissolved in recording solution and applied using a SF-77B fast perfusion system (Warner Instruments) at a rate of 1 ml/min.

### Site-directed Mutagenesis

Point mutations on pcDNA3-rP2X4 (K362Q-K363Q) and pcDNA3-rP2X5 (S365K-E366Y-E374K) were introduced using the QuikChange mutagenesis method (Stratagene).

### Phospholipid-binding Assay

Oligonucleotide-based sequences coding for P2X C-terminal sequences (P2X1: L352-E378, P2X4: C360-V375, P2X5: L361-V376, P2X7: P376-I391, P376-P396, A378-E393) were subcloned into the pGEX-2T vector for the production of GST fusion proteins. Binding assays were conducted on phospholipid-coated hydrophobic membranes (PIP Strips™, Echelon Biosciences). GST fusion proteins (1 µg/ml) were applied overnight, primary (mouse anti-GST, 1∶1000) and secondary (goat anti-mouse HRP, 1∶5000) antibodies were applied for one hour. All washes and protein or antibody incubations were done in TBS+T solution supplemented with 3% BSA. Bound proteins were detected by ECL (PerkinElmer).

### Data Analysis

Peak currents, defined as the maximal amplitude recorded during agonist application, were measured. For current rundown, the amplitude of the second response (after a 4-minute wash) was compared to the first and expressed as a percentage. For current kinetics, activation rate was measured as the rise time (in seconds) from 10% to 90% of the peak amplitude. For desensitization rate, the 5-second decay % was used for P2X4 and the decay slope was measured for P2X5. Data are presented as mean ± SEM. Statistical analyses for the difference in means were carried out using Student’s *t* test for two unpaired groups, one-way or two-way ANOVA followed by a Bonferroni post-test.
